# Uncommon BK polyomaviruses detected in allogeneic hematopoietic cell transplant recipients

**DOI:** 10.1128/mra.00005-24

**Published:** 2024-04-22

**Authors:** Saagar M. Chokshi, Elizabeth A. Odegard, Heidi L. Meeds, Steven B. Kleiboeker, Assem Ziady, Anthony Sabulski, Sonata Jodele, Alix E. Seif, Stella M. Davies, Benjamin L. Laskin, Jason T. Blackard

**Affiliations:** 1 Division of Digestive Diseases, University of Cincinnati College of Medicine, Cincinnati, Ohio, USA; 2 Eurofins Viracor Laboratories, Lenexa, Kansas, USA; 3 Department of Pediatrics, University of Cincinnati College of Medicine, Cincinnati, Ohio, USA; 4 Division of Bone Marrow Transplantation and Immune Deficiency, Cincinnati Children’s Hospital Medical Center, Cincinnati, Ohio, USA; 5 Perelman School of Medicine, University of Pennsylvania, Philadelphia, Pennsylvania, USA; 6 Division of Oncology, Children's Hospital of Philadelphia, Philadelphia, Pennsylvania, USA; 7 Division of Nephrology, Children's Hospital of Philadelphia, Philadelphia, Pennsylvania, USA; Katholieke Universiteit Leuven, Leuven, Belgium

**Keywords:** BK polyomavirus, hemorrhagic cystitis, hematopoietic cell transplant, viral diversity, genotype

## Abstract

The role of viral diversity in the pathogenesis of BK polyomavirus (BKPyV)-associated disease is poorly understood. Here, we report near full-length BKPyV genome sequences from two allogeneic hematopoietic cell transplant recipients infected with BKPyV genotype II, which is uncommon in the USA.

## ANNOUNCEMENT

BK polyomavirus (BKPyV) is a double-stranded circular DNA virus in the family *Polyomaviridae* and genus *Betapolyomavirus* (1). BKPyV infects over 80% of the world’s population, typically in childhood, and persists in a state of minimal replication (1, 2). Immunosuppression permits viral reactivation, which can lead to BKPyV-associated hemorrhagic cystitis in hematopoietic cell transplant (HCT) recipients and BKPyV-associated nephropathy in kidney transplant recipients (3). The impact of viral diversity on the pathogenicity of BKPyV is poorly understood. Here, we report near full-length BKPyV genome sequences from two allogeneic HCT recipients infected with BKPyV genotype II. Genotype II is not observed commonly in the USA, and limited genotype II genome sequences have been reported globally (4).

Subject 1 is an 11-year-old male from the United Arab Emirates with Glanzmann thrombasthenia who underwent a matched unrelated donor allogeneic HCT after myeloablative conditioning with busulfan and cyclophosphamide. BKPyV viremia was detected on day 38 (1,455 copies/mL) but was not detected at any other timepoints. BKPyV viruria was not measured clinically as the patient did not develop cystitis symptoms.

Subject 2 is a 4-year-old African American male from Ohio with sickle cell disease who underwent a matched sibling allogeneic HCT after reduced-intensity conditioning with alemtuzumab, fludarabine, and melphalan. BKPyV viremia was first detected on day 31 after HCT (559 copies/mL) and reached a maximum plasma level of 10,606 copies/mL on day 80. Urine BKPyV was first measured with cystitis symptom onset on day 38 after HCT and was >20 million copies/mL.

**Fig 1:**
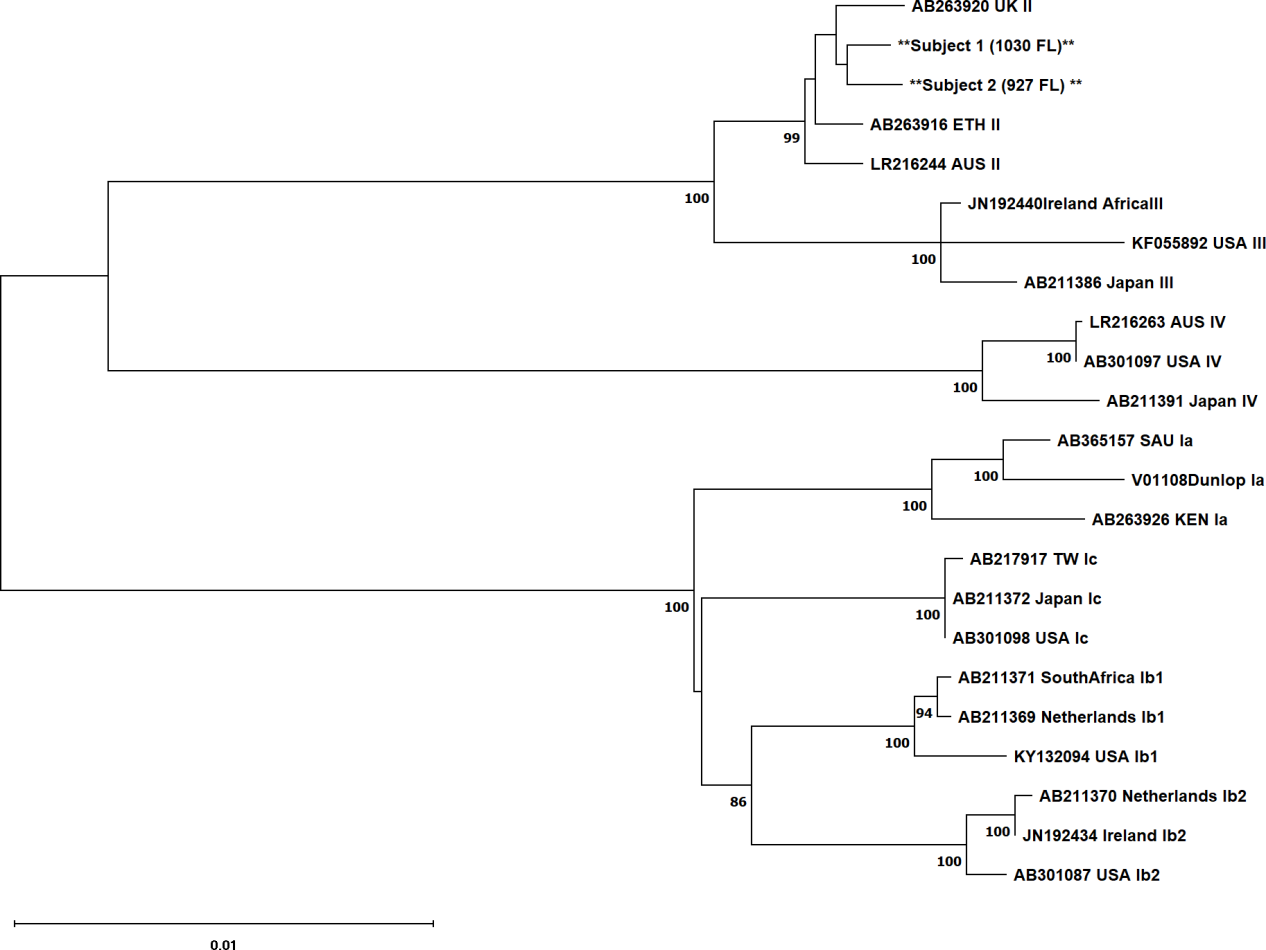
Phylogenetic tree generated with ClustalX 2.1 and visualized in Mega 11. References are identified by accession number, country of origin, and genotype. Samples are indicated with flanking asterisks. The scale bar represents 0.01 nucleotide substitutions per site. Bootstrap values ≥70 included as node labels.

BKPyV viremia and viruria were detected and quantified at Eurofins Viracor laboratory (3). BKPyV DNA was extracted and amplified from urine samples obtained 1 month post-HCT using a published protocol (5). Briefly, viral DNA was extracted from 1 mL of urine using the Qiagen QIAmp UltraSens Virus Kit. Circular DNA was non-specifically amplified via rolling circle amplification and then linearized. The near full-length BKPyV genome was amplified via polymerase chain reaction (PCR) using the BK1731F and BK1739R primers. PCR products were run on a 1% agarose gel, extracted, and sequenced using next-generation sequencing (NGS). Libraries were prepared using the NEBNext Ultra II FS DNA Library Prep Kit and sequenced under the setting of PE 2 × 61 bp on an Illumina NextSeq 2000. NGS reads were run through FastQC to assess quality control, and no reads were flagged as poor quality. All tools were run under default parameters. Reads for each sample were mapped to a GenBank reference genome (V01108 [Dunlop]) using UGENE 48.1 and the Bowtie2 mapping tool to generate a consensus sequence with near complete genome coverage for each subject. Details regarding raw reads and consensus sequences are presented in Table 1. Consensus sequences were aligned via multiple sequence alignment in ClustalX 2.1 with GenBank references of known BKPyV genotypes. A phylogenetic tree was constructed from the multiple sequence alignment in ClustalX 2.1 using the neighbor-joining method and visualized in Mega 11 (Fig. 1). Samples were determined to be genotype II based on clustering with genotype II references. Pairwise genetic distances were calculated in Mega 11 using the Kimura 2-parameter model. There is a 99.77% nucleotide similarity between the Subject 1 and Subject 2 sequences. There is a 99.70% nucleotide similarity between the Subject 1 sequence and its closest related GenBank reference sequence (AB263920 UK II) and a 99.68% nucleotide similarity between the Subject 2 sequence and its closest related GenBank reference sequence (also AB263920 UK II).

**TABLE 1:** BKPyV genome characteristics

Subject	Raw reads		Consensus sequences	
	Number of reads	Average read depth	Length	G/C content
1	5,369,786	63,566	5,153 bp	39.36%
2	2,581,833	30,563	5,153 bp	39.43%

## Data Availability

Raw sequence data are available under BioProject PRJNA670723 with SRA accession numbers SRX22222802 for Subject 1 and SRX22222801 for Subject 2. Consensus sequences are available in GenBank with accession numbers OR734229 for Subject 1 and OR734228 for Subject 2.
